# From Formamide to RNA, the Path Is Tenuous but Continuous

**DOI:** 10.3390/life5010372

**Published:** 2015-01-30

**Authors:** Samanta Pino, Judit E. Sponer, Giovanna Costanzo, Raffaele Saladino, Ernesto Di Mauro

**Affiliations:** 1 Fondazione “Istituto Pasteur-Fondazione Cenci-Bolognetti” c/o Dipartimento di Biologia e Biotecnologie “Charles Darwin”, “Sapienza” Università di Roma, P.le Aldo Moro, 5, 00185 Rome, Italy; E-Mail: samantapino78@libero.it; 2 Institute of Biophysics, Academy of Sciences of the Czech Republic, Královopolská 135, 61265 Brno, Czech Republic; E-Mail: judit@ncbr.muni.cz; 3 CEITEC-Central European Institute of Technology, Masaryk University, Campus Bohunice, Kamenice 5, CZ-62500 Brno, Czech Republic; 4 Istituto di Biologia e Patologia Molecolari, CNR, P.le Aldo Moro, 5, 00185 Rome, Italy; E-Mail: giovanna.costanzo@uniroma1.it; 5 Dipartimento di Scienze Ecologiche e Biologiche Università della Tuscia Via San Camillo De Lellis, 01100 Viterbo, Italy; E-Mail: saladino@unitus.it

**Keywords:** formamide, nucleic bases, uridine, acyclonucleosides, 3',5' cyclic nucleotides, RNA, RNA polymerization, RNA origin, ribozymes, origin of life

## Abstract

Reactions of formamide (NH_2_COH) in the presence of catalysts of both terrestrial and meteoritic origin yield, in plausible and variegated conditions, a large panel of precursors of (pre)genetic and (pre)metabolic interest. Formamide chemistry potentially satisfies all of the steps from the very initial precursors to RNA. Water chemistry enters the scene in RNA non-enzymatic synthesis and recombination.

## 1. Introduction

Top-down reasoning is the most direct, logical approach to biological problems related to systems and processes that are not occurring anymore. This is especially true when one deals with origin of life matters. In reasoning and experimenting about RNA origins, two different, but potentially converging, approaches are used: the pragmatic and the philological.

The first uses straight-to-the-purpose experimental approaches and systems: e.g., highly pre-activated precursors, preformed sequences, ingenuous laboratory procedures. The results are actually and potentially extremely relevant. As an example, the use of highly-activated precursors as phosphoramidated nucleotides has allowed unprecedented progress in* in vitro* molecular evolution studies [1,2,3,4,5,6,7,8,9,10] and is fostering the development of protocells in which non-enzymatic RNA replication and evolution is possible [11]. Recent progress towards an RNA replicase ribozyme [12,13,14] is impressively close to a possible reconstruction of the “novel chemistry that life brought to Earth” [15]. A similar, conceptually empirical approach has led to the *in vitro* construction of a nucleoside [16]. The answers obtained are there to stay.

The philological approach is, on the contrary, full of hurdles and prone to frustrating obstacles. Since the components of the earliest probiotic systems can only be hinted at, experimental set-ups are very difficult to optimize. Optimization uses *a posteriori* logic and uses the results of evolution to inspire the experimentalist.

Philology aims to reconstruct those processes whose components remain largely unknown. The only clue for the philological experimentalist is: (1) Occam’s razor (the simpler, the more likely); and (2) we know the result we are aiming for—an autogenerated RNA that displays ribozyme activity and that can be integrated into active metabolic machineries that harness and redistribute energy. Can one dare to undertake a philological endeavor in the form of a bottom-up approach?

## 2. The First Part of the Road Is Outlined

We reasoned that robust syntheses require robust precursors. The purport of this word translates into “abundant”, “stable” and “reactive”. The astronomically most abundant carbon-containing, three-atom compound is hydrogen cyanide (HCN), whose reactivity, in the presence of water, leads to formamide (NH_2_COH), a compound that is stable in liquid form between 4 and 210 °C, but it still keeps some reactivity. Our interest was attracted by these properties and by the fact that, with a few exceptions [17,18], its prebiotic relevance in the past century was largely overlooked.

**Figure 1:**
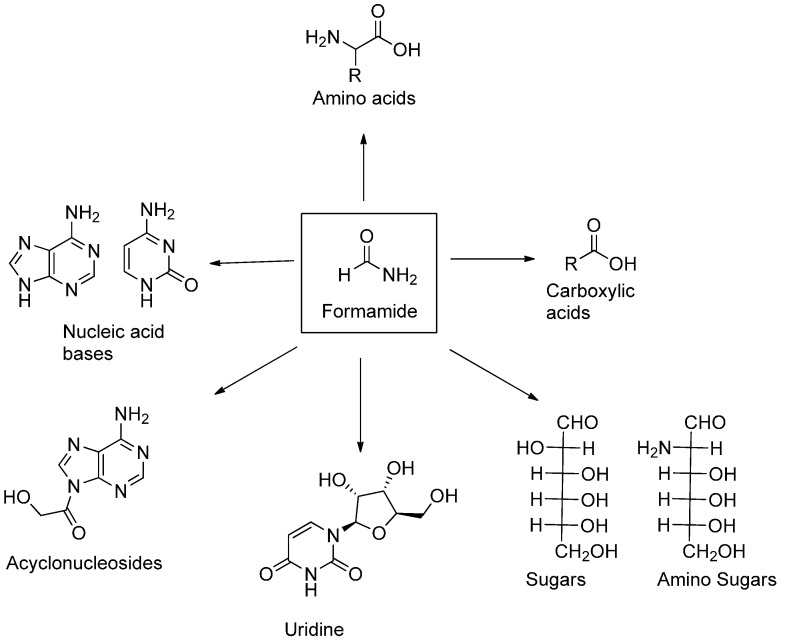
Syntheses from formamide (see [20,21,22,25]).

**Figure 2:**
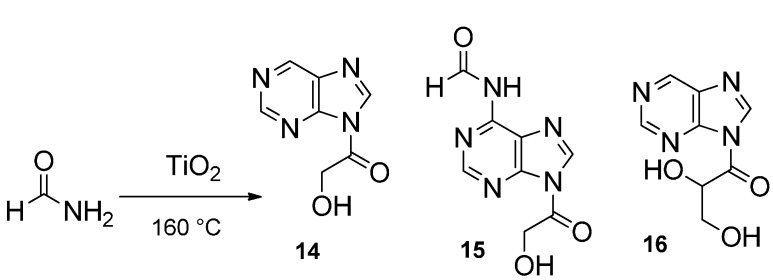
Synthesis of acyclonucleosides from formamide (see [20,23]).

One notable exception is the studies by Yamada* et al.*, who, in 1978, reported [17,18] that heating liquid NH_2_COH at 160 °C yields low amounts of purine, while adenine can be synthesized in traces when the reaction is performed in the presence of added HCN [17]. These same authors showed [18] by nuclear magnetic resonance experiments that three equivalents of HCN and two of NH_2_COH are embedded in the heterocyclic ring through a C-N bond fission process. By heating formamide (100–160 °C) in the presence of simple catalysts, we observed that large panels of prebiotically relevant compounds could be obtained. An analytical effort started in 2001 [19], which has been pursued to the present day, thoroughly examining the effect on formamide-based, prebiotically relevant syntheses of a large spectrum of catalysts encompassing terrestrial and meteorite minerals. As for the terrestrial mineral-based catalysts, the products obtained and the numerous detailed chemical aspects of these syntheses were recently reviewed [20,21] (Figure 1). The products obtained in the presence of 12 different meteorites were also reported [22], detailing non-fastidious syntheses of all the extant RNA and DNA nucleic bases, 13 amino acids, 18 carboxylic acids, including a solid representation of the components of the TCA cycle, and condensing agents, such as carbodiimide-urea. In particular, acyclonucleosides were also obtained [23] (Figure 2). The energy sources tested were thermal (as reviewed in [20,21]), various wave lengths [23,24] of light and high-energy irradiation. A partial account of the syntheses from formamide performed with irradiation by a 165-MeV proton beam at the Dzelepov Laboratory of Nuclear Problems (DLNP) Phasotron (4 min at a dose rate of 1.5 Gy/min) in Dubna (Russia) has appeared [25] and has reported that in the presence of the stony iron meteorite, North-West Africa NWA 4482, as a catalyst, the nucleoside, uridine, is synthesized (yield = 0.54 μg/1.5 mL formamide) (Figure 1), along with a large panel of other compounds.

In summary, the route from [HCN + H_2_O] to H_2_NCOH, all the way to a large set of prebiotic compounds of (pre)genetic and (pre)metabolic relevance appears to be, at the moment, an essentially continuous path, devoid of interruptions or thermodynamically or kinetically insurmountable gaps. Naturally, this is not to say that the scenario is totally clear or complete, especially in the absence of an agreed-upon definition of the ur-environment. The route from formamide to acyclonucleosides and to at least one extant nucleoside is well-understood and continuous, recently gaining consensus [26,27]. In formamide and in the presence of a phosphate source [28,29,30,31], preformed nucleosides are phosphorylated in every possible position (2',3' and 5'), affording the synthesis of 2',3'- and 3',5'-cyclic nucleotides.

## 3. Nonenzymatic RNA Polymerization from 3',5' Cyclic Nucleotides

Polymerization of 3',5'-cAMP and of 3',5'-cGMP has been reported [32]. Polymerization of 3',5'-cGMP in water was partially characterized [32,33,34], showing that spontaneous polymerization of 3',5'-cyclic GMP occurs in water, or in formamide, or in dimethylformamide and is stimulated (in water) by Brønsted bases, such as 1,8-diazabicycloundec-7-ene. The reaction is untemplated, does not require enzymatic activities, is thermodynamically favored and selectively yields 3',5'-bonded ribopolymers containing as many as 25 nucleotides. The reaction products were analyzed by denaturing PAGE, MALDI ToF MS [31], P-NMR and analysis by specific RNases of the polymerized materials.

**Figure 3:**
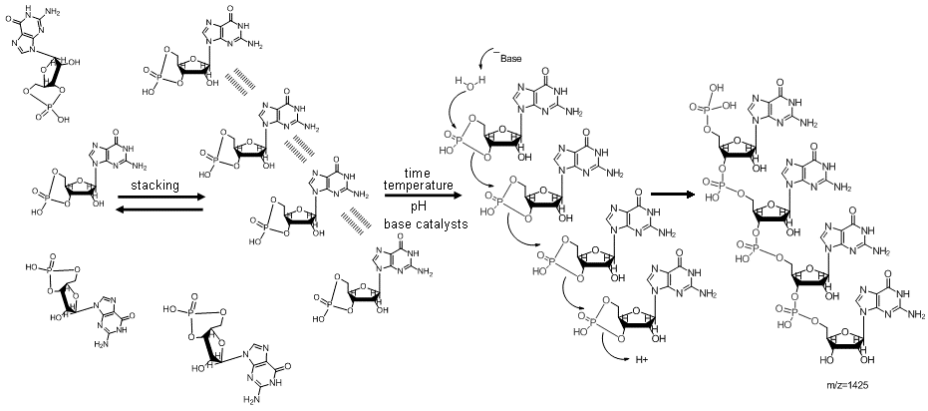
The mechanism of polymerization of 3',5'-cGMP (see [33]).

Based on the measured stacking of the 3',5'-cyclic monomers [33], the activation of the reaction by Brønsted bases and the determination of the molecular species produced, a reaction pathway was proposed [33] consisting of a simple process based on the formation of base stacking-supported pillared structures, followed by position-stimulated polymerization by trans-phosphorylation (Figure 3).

This polymerization reaction has been confirmed by Dieter Braun’s group [35], showing that the reaction may also occur under dry conditions. In this case, the product was analyzed by *a posteriori* intercalation of the fluorescent dye, SYBR Gold. The detection of polymerization by this additional and different technique validates the reaction.

Polymerization of 3',5'-cGMP is more efficient under dry conditions than in water; the difference consisting in (i) faster kinetics and (ii) a lower temperature requirement for the transphosphorylation step under dry conditions (to be detailed elsewhere). Enhancement of polymerization by decreasing water concentrations coheres with the Gibbs free-energy paradox, which states that reactions that proceed through the release of water are hampered by the presence of water [36,37].

**Figure 4:**
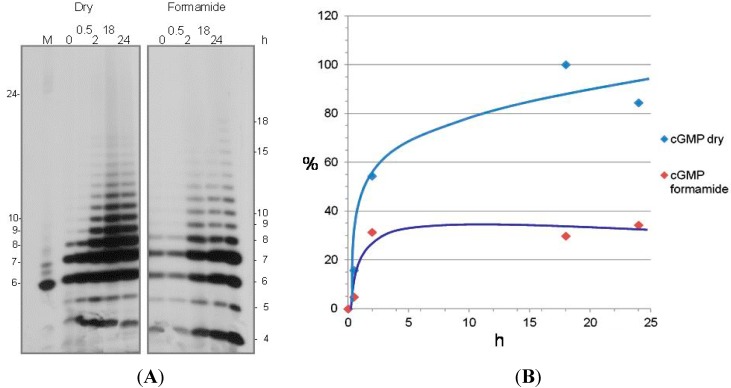
Polymerization of 3',5'-cGMP under dry conditions and in formamide. (**A**) Autoradiogram of gel electrophoretic analysis of the polymerization products of 3',5'-cyclic GMP. Polymerization, terminal labelling and analysis were performed essentially as described in [32,33,34], with the specification that the reaction was carried out under dry conditions at 60 °C for the time spans (in hours) indicated on top of each lane (left) or in 100 μL of pure formamide (right). The reactions was performed using 3',5'-cGMP Na^+^-free, H^+^ form (custom made by BioLog LSI, Bremen, Germany), which was neither evaporated nor precipitated during the preparation steps, concentrated from the initial 1 mM concentration in MilliQ water by evaporation in Savant under vacuum and cooling mode till dryness was obtained. The length of the oligomers is indicated in nucleotides on the sides. (**B**) Quantitative analysis of the polymerization products. Data from (A) are expressed as % obtained in each condition relative to the maximum synthesis observed (18 h under dry conditions).

The comparison of the polymerization of 3’,5’-cGMP under dry conditions and in formamide (Figure 4) shows that: (i) polymerization occurs in both conditions; with (ii) comparable efficiency and kinetics; (iii) non-enzymatically and non-templatedly yielding oligomers.

Again, this is not to say that RNA non-fastidiously and easily emerged out of a formamide solution. However, the observations that: (i) nucleoside synthesis is streamlined when initiated by formamide even though under high-energy proton beams [25] mimicking perhaps space or very early unshielded Earth conditions, (ii) preformed nucleosides are phosphorylated in formamide [28,29,30,31] yielding cyclic derivatives [30,31] and (iii) that at least purine cyclic nucleotides undergo polymerization, indicate that from formamide to RNA, the path is in principle plausible.

In thermodynamical terms, RNA polymerization is a form of stabilization, as detailed by the comparative analysis of the stability of the weakest bonds in RNA precursor monomers or in RNA [38,39] (and, for that matter, also in DNA [40]). A detailed analysis of ribonucleoside stability was reported [39]. From a Darwinian perspective, in a prebiotic world, this property could have provided a pre-cellular advantageous phenotype, leading to the favored accumulation of nucleosides/nucleotides in the more stable polymeric form.

## 4. RNA Shows Properties of Auto-Catalytic Reactivity, Even in Its Simplest Sequences

Many models propose [41,42,43] that in order to allow prebiotic evolution, RNA molecules had to transfer the information encoded by their nucleotide sequence to other molecules, fostering information complexity. This process would have subsequently evolved RNA into molecules endowed with the catalytic functions necessary for the reproduction of sequence information. The theoretical scheme from astrochemical reaction products to long RNAs exhibiting catalytic activity has been thoroughly discussed [44,45], but progress in defining simple RNA sequence exchanges that could apply to plausible prebiotically simple scenarios has been slow. *In vitro* RNA evolution studies are a highly developed domain [46,47,48], having established both principles and methodologies from a given level of complexity upwards. The primordia of these processes remain uncertain. An important progress was reported showing the nonenzymatic recombinations of RNA by means of transesterification ([49] and the references therein). Particularly relevant to this topic is the pioneering work of the group of A. Chetverin [50,51]. An experimental system developed by Vlassov’s group ([49,52] and the references therein) consisted of coupled nonenzymatic cleavage/ligation of oligonucleotides catalyzed by magnesium ions, affording longer RNA molecules with a new sequence. Still, it started from pre-synthesized, relatively complex RNA sequences. With the possible exception of the systems described in [52,53,54], the reactions reported so far [46,47,48,55,56,57,58] are of interest to a relatively advanced evolutionary stage.

Of even greater interest is the observation [34] that when reacted with fully or partially sequence-complementary RNA (oligoC), the abiotically generated oligoG RNA [32,33,34] displays a typical ribozyme activity consisting of terminal ligation accompanied by cleavage of an internal phosphate site of the donor oligonucleotide stem upon attack of the acceptor 3' terminal OH. This reaction is dubbed ligation following intermolecular cleavage (LIC) and was observed only for those oligoC/oligoG combinations that underwent ligation, as well. From a prebiotic perspective, the ability of oligoG polynucleotides to react with other sequences in a manner other than ligation outlines a simple and possible evolutionary scenario based on the autocatalytic properties of RNA.

In the LIC reaction, the two complementary sequences assist each-other’s cleavage and terminal recombination. In the cleavage reaction, the 3' OH extremity of each sequence behaves (in ribozyme terminology) [59] as an “acceptor”. In the same terminology, “donor” refers to the phosphate donor site. This reaction suggests the existence of a recombination mechanism between complementary sequences resulting in RNA chain elongation by addition at the 3' extremity in simple RNAs.

**Figure 5:**
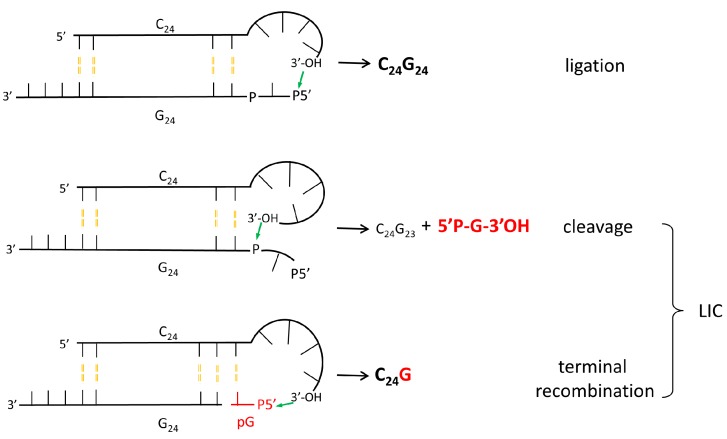
The ligation following intermolecular cleavage (LIC) mechanism. The reaction between C_24_ and 5'-phosphorylated G_24 _is shown as an example. Ligation assuming loop formation at the 3'-end of C_24_ and attack at the phosphorylated 5'-end of G_24_ leads to the formation of C_24_G_24_. Simultaneous cleavage reaction initiated by the attack of the 3'-end of C_24_ at the penultimate phosphate of the 5'-phosphorylated G_24_. The products of this reaction are C_24_G_23_ and 5'-phosphorylated guanosine-phosphate, which readily combines with another C_24_, leading to the formation of C_24_G (see [34]).

**Figure 6:**
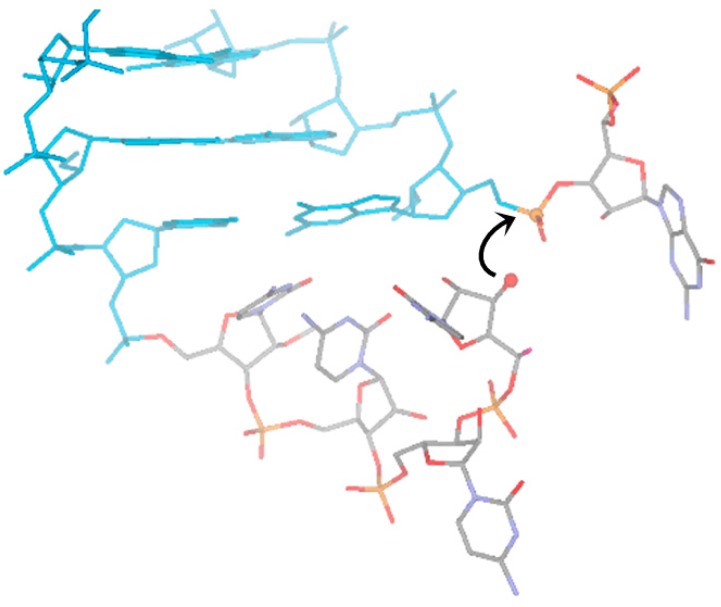
Ribozyme activity of spontaneously-generated RNA. 3D model of a plausible loop geometry that initiates the cleavage of the 5'-terminal nucleotide of the donor oligonucleotide strand. The direction of the in-line attack is shown with a black arrow, the four nucleotides of the unpaired loop-like overhang are colored according to the constituting atoms, while the Watson–Crick paired segment is colored in blue (see [34]).

The prebiotically relevant point here is that this reaction occurs by the action of spontaneously-generated polymers derived from abiotically plausible precursors, enhancing the information content of a polymeric mixture. This mechanism potentially represents a plausible means to generate RNA sequence complexity and to approach the questions: how could the extremely complex ribosomal machinery [60] start to evolve, and how did the initial RNA transfer functions [61] come about? The proposed mechanism for the LIC reaction is described in Figure 5 and Figure 6. As observed [34], Watson–Crick-type base-complementarity is needed to achieve self-cleavage of the interacting oligomers. In order to put the ligation and cleavage reactions into a common frame and fulfill the requirements of Watson–Crick complementarity, the two interacting strands are ideally shifted in register by 3–4 bases (Figure 5), enabling the formation of a loop at the 3'-end of the acceptor strand. This loop formation is necessary to bring the 3'-end into an in-line attacking position at the 5'-phosphorylated end of the donor strand (Figure 5). At the same time, the loop can easily adopt also a geometry in which the 5'-phosphorylated end is attacked at the penultimate phosphate group (Figure 5), which becomes accessible to nucleophilic attack due to end-fraying. These considerations suggest that Watson–Crick base pairing combined with the ability to form stable loop geometries could provide the structural basis of the catalytic activity of the first RNA-oligomers. Based on detailed computations of the free energy profiles of the ligation and cleavage reactions, a consistent model for these reactions that lead to terminal RNA recombination has been formulated (Figure 6) [34].

Summarizing these studies, RNA molecules derived from the interactions of non-enzymatically polymerized oligoG with oligonucleotides containing a sufficiently long complementary sequence are active ribozymes, their activity consisting of the nucleophilic attack of an acceptor 3'-hydroxyl group on the phosphorus of a donor 3',5' phosphodiester bond. The location of the donor phosphate, the orientation and the structure of the surrounding sequences determine the result, consisting of a single cleavage, leading to a one-step chain elongation event.

The cleavage by the 3' OH of the acceptor molecules preferentially occurs on an XpX step located one step 3' distal from the 5' extremity, in an unpaired structure, at the tip of the presumptive sequence complementarity-determined double-stranded structure (Figure 5), presumably because the unpaired tip conformation allows local sterical availability (end-fraying). Changing the nature of the tip by insertion of increasing long stretches of residues at the potential interaction site did not modify the cleavage position. Thus, in all of the analyzed instances, the donor site is the XpX step at the first unpaired position at the terminus of a double-stranded RNA.

The fact that the interaction between simple sequence oligonucleotides leads to further transformation of the sequence information provides the proof-of-principle that the nonenzymatic generation of oligoG RNA molecules from the prebiotically plausible 3',5'-cyclic nucleotides is the first step of a potentially (pre)genetic process.

No matter how complex the variety of extant ribozyme activities are, they necessarily evolved from initially simple sequences [62,63] endowed with the appropriate chemical potentiality. The data reported here suggest that the properties of the G sequence might have been instrumental in this process.

## 5. Conclusions

We have here concisely described that all of the steps leading from formamide to short RNA sequences endowed with simple ribozyme activity are experimentally plausible. Life is a robust phenomenon, and its origin most likely depended on robust processes, easily available starting materials and thermodynamically sound reactions. Formamide chemistry might have played a non-secondary role in origin-of-life processes: its abundance in space has been recently reported [64]; the numerous reactions leading to its formation both in space- and in Earth-wise conditions and the properties that might have favored its concentrations are known in detail [20,21]. The reactions described here occur in moderate, prebiotically plausible conditions. Taken together, these reactions draw a thin, but continuous line from a one-carbon compound to actively reactive (pre)genetic molecules.

## Figures and Tables

**Figure A1:**
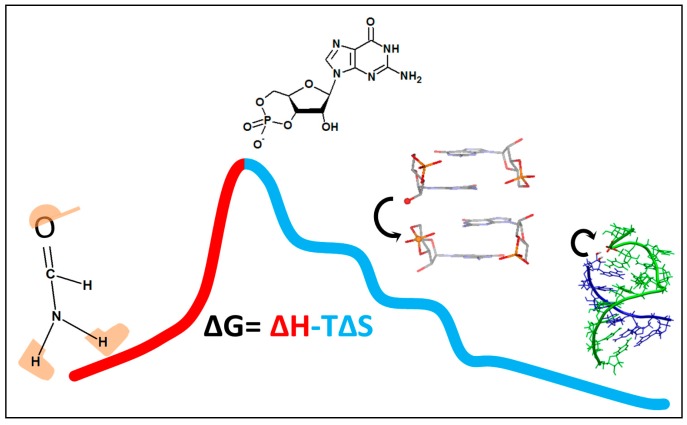
The great track of formamide towards the first genetic molecules: cyclic nucleotides synthesized from formamide using thermal energy spontaneously self-assemble to give short oligomers. In the next step, imperfect pairing between WC-complementary strands and the transient formation of tetraloop-like overhangs could lead to the emergence of an ancient form of RNA-catalysis.
